# The SINS trial: A randomised controlled trial of excisional surgery versus imiquimod 5% cream for nodular and superficial basal cell carcinoma

**DOI:** 10.1186/1745-6215-11-42

**Published:** 2010-04-21

**Authors:** Mara Ozolins, Hywel C Williams, Sarah J Armstrong, Fiona J Bath-Hextall

**Affiliations:** 1 Centre of Evidence-Based Dermatology, King's Meadow Campus, Lenton Lane, Nottingham, NG7 2NR, UK; 2 Design Service for the East Midlands (NDL) 14th Floor, Tower Building, University Park, University of Nottingham, Nottingham, NG7 2RD, UK

## Abstract

**Background:**

Basal cell carcinoma is the commonest human cancer. Despite increasing incidence it remains poorly researched. While not life threatening it can cause significant cosmetic disfigurement. Imiquimod, a cream which enhances the body's immune response, may help deal with the number of cases that occur in low-risk sites, especially when good cosmetic results and home use without surgery are needed.

This study aims 1. To compare excisional surgery with imiquimod cream for nodular or superficial basal cell carcinoma in low risk sites, with respect to 3 year clinical clearance, cost-effectiveness and cosmetic results. 2. To ascertain if certain phenotypic features and gene polymorphisms predict tumour responsiveness to treatment.

**Methods/Design:**

Five hundred participants with low risk nodular or superficial basal cell carcinoma will be recruited from hospitals to this multi-centre, randomised, parallel group, controlled phase III trial. Treatment in the imiquimod group is for 6 weeks for superficial basal cell carcinoma and 12 weeks for nodular basal cell carcinoma. Both treatment groups are followed up in clinic for 3 years. Primary outcome variable: the proportion of participants with clinical evidence of success (no recurrence) at 3 years. The primary outcome will be compared between the two treatment groups. Secondary outcomes include: i) clinical success at 1, 2 and 5 years, ii) time to first recurrence, iii) cosmetic appearance of lesion site after treatment, iv) level of pain, and v) cost-effectiveness. Safety and tolerability data will also be reported.

**Discussion:**

This study protocol describes a pragmatic randomised controlled trial which it is hoped will address the above uncertainties. Three-year results will be available towards the end of 2010.

**Trial registration:**

Meta-register: NCT00066872, Eudract No. 2004-004506-24, ISRCTN48755084.

## Background

Basal cell carcinoma (BCC), or "rodent ulcer" is the commonest human cancer [1-3] affecting at least 30,000 UK people annually, with increasing incidence [4], especially in younger people [5] and in those with higher social class [6]. Although not life threatening, BCC can cause significant cosmetic disfigurement, and remains one of the most poorly researched human cancers [7]. Most BCCs are treated surgically in hospitals, but current treatment provision is becoming saturated in many centres in the UK. A systematic review highlighted topical imiquimod as a potentially useful therapy for superficial basal cell carcinoma and raised the possibility that it could also be useful for dealing with the bulk of low risk nodular basal cell carcinomas - thereby releasing limited surgical resources in the UK dermatology centres to tackle more complicated tumours at high risk sites.

Imiquimod cream enhances the body's immune response. Although not as effective as surgery, it may result in a better cosmetic result (potentially important as a majority of BCCs appear on the face and neck [8,9]) together with the added convenience of home application.

Prior to this study, imiquimod cream was not used routinely for BCC, and clinical opinion was divided regarding its potential usefulness for different types of BCC. It is thus timely to consider these issues to prevent assimilation of practice without adequate evaluation.

If imiquimod 5% cream has an acceptable success rate, is cost-effective and easy to use, then it could be an effective treatment option for the routine first treatment of the majority of low risk nodular and superficial BCCs seen in skin cancer clinics in the UK and elsewhere. Recurrences and high risk BCCs could then be dealt with surgically by dermatologists and plastic surgeons working as part of the skin cancer teams. Treatment with imiquimod could even start in the community after histological diagnosis, using a strict protocol to prevent indiscriminate use (and diagnostic uncertainty and/or possible delay in melanoma diagnosis) and hence increase in costs.

## Methods/Design

### Trial objectives

We seek to answer the following questions:

1. Can imiquimod 5% cream applied topically give an acceptable and clinically useful success rate (3 year clinical clearance) and acceptable side effect profile when compared with excision surgery for superficial and nodular BCC at low risk sites?

2. Is imiquimod more cost effective than surgery for low-risk BCC?

3. Does imiquimod result in a more aesthetically acceptable result than conventional excision?

4. Do certain phenotypic features and gene polymorphisms predict tumour responsiveness to treatment?

We hypothesise that the clinical success rate at 3 years among participants treated with imiquimod cream will not be inferior to the clinical success rates obtained from surgery.

Both nodular and superficial BCC are included in this trial because in real life clinicians are unlikely to make an *a priori *decision on what constitutes a nodular and superficial lesion, and even if they do, their clinical decision may be wrong (compared to histology, which is also somewhat arbitrary). The agreement between clinical and histopathological diagnosis will be looked at.

Long-term follow-up is needed to catch late recurrences of slower-growing tumours [10-13]. There is a real danger that lesions that appear clear in the short term e.g. at 1 year, will recur in the long term. It is also unclear whether the short-term histological clearance reported in the Phase II studies can be translated into a durable clinical clearance, and there may be a gradient of treatment response depending on the histological subtype. More precise estimates of treatment efficacy using much larger sample sizes are needed for the commoner nodular BCC. A definitive independent trial with cost-effectiveness data and long term follow up will help to inform patients, health care workers and the NHS. Information on possible predictors (clinical and genetic) of treatment response might also be useful for guiding clinical practice.

### Trial design

Prospective, multi-centre, randomised, parallel group, controlled phase III trial, to compare excision surgery and imiquimod cream for nodular and superficial basal cell carcinoma presenting in low risk areas [3]. This is a simple pragmatic study [14].

The consultant dermatologist will decide if the lesion is superficial or nodular at the study outset (confirmed by the diagnostic biopsy result).

### Trial interventions

1. Imiquimod 5% cream once daily for 6 (superficial - clinically diagnosed) or 12 (nodular) weeks total, or

2. Surgical excision with a 4 mm margin (accepted standard at trial set-up). The surgical group will be operated on as soon as local conditions permit.

Treatment failures (after either a biopsy or a period of watchful waiting) will be treated by surgical excision. Treatment duration and frequency of dosing are based on results of existing studies [15] at study set-up, and a balance of efficacy with effort for the participants and possible side effects. The dosing frequency was unchanged after the manufacturers presented results from a large dose-finding clinical study to us, however after the start of this study imiquimod became licensed for superficial BCC, with a 5 days per week dosing frequency.

In order to identify possible genetic markers for tumour response, 5 ml of blood will be taken from consenting participants. Methods for analysing the genetic marker data are given in a separate genetic markers addendum to the protocol.

### Treatment adherence

In the imiquimod group local reactions and the long duration of daily treatment are factors which could compromise compliance. The application of the cream will be explained. Cards for monitoring daily pain will be given to participants in both groups, and returned every six weeks. A telephone help-line will be available for answering day to day problems. Participants will be asked to return used and unused imiquimod sachets (one per day of treatment).

### Participant selection

At each of 12 (initially three) hospitals potentially suitable participants will be asked to speak to the research nurse, who explains the study, checks eligibility and deals with informed consent. Entry criteria are as broad and inclusive as possible in order to approximate the sort of patients encountered outside the usual strict clinical trial environment and hence to improve external validity.

#### Inclusion criteria

• Men and women of any age who present clinically with either primary nodular or superficial BCCs, or a skin lesion other than BCC which turns out on histology to be a superficial or nodular BCC

• Any number of BCCs (although only one per participant is selected for the study)

• Histologically proven BCC (usually a punch or shave biopsy specimen of no more than 25% of the total lesion, though sometimes at surgery)

• Location of primary nodular/superficial BCC in low risk areas (avoiding use of imiquimod in areas at high risk of recurrence where delay of appropriate surgery might result in more invasive procedures)

• Informed consent

• Must have access to a telephone

#### Exclusion criteria

• Genetic or nevoid conditions such as Gorlin's syndrome

• Morphoeic (microinfiltrative) trial lesion as diagnosed clinically (even if not histologically classed as morphoeic - histological sample may have missed infiltrative nature of lesion)

• Allergy to any of the interventions

• Involvement in a trial of another experimental intervention

• Life threatening disease

• Bleeding disorders

• Not available for follow up for 3 yrs

• Pregnant, intention to become pregnant during treatment phase of the trial, or breastfeeding

### Outcome measures

#### Primary

The proportion of participants with clinical evidence of "success" (defined as absence of treatment failure or any signs of local recurrence) at 3 years as judged by a consultant dermatologist, as would occur in routine clinical practice. Participants allocated cream, that subsequently require surgery for poor response or recurrence will be counted as treatment failures, if histology is proven to be positive.

#### Secondary

i) The proportion of participants with clinical success at one, two and five years.

ii) Time to first recurrence (classified into one of the following 5 categories according to the period between follow up visits when this occurred: "prior to year 1 visit"; "between year 1 visit and year 2 visit"; "between year 2 visit and year 3 visit"; "between year 3 visit and year 5 visit"; "no recurrence prior to year 5 visit"). The actual time of recurrence is not known, but only the visit at which it is diagnosed.

iii) The proportion of participants with a lesion of excellent or good appearance at 6 months and at 3 years. (The cosmetic appearance of a participant's lesion will be rated independently by the participant and blinded observer. using a five point scale ("Excellent", "Good", Fair", Poor", "Very poor")).

iv) The proportion of participants experiencing a moderate or more severe level of pain a) during treatment and b) in the 16 weeks following treatment. (This will be measured daily using a six-point scale ("No pain", "Mild", "Mild-Moderate", "Moderate", "Moderate-Severe", "Severe") using a questionnaire completed at home).

v) Cost effectiveness to include number of participant visits to hospital, as well as cost of treatment per session.

Clinical clearance will not be routinely confirmed by further biopsy mainly because a) this would not happen in clinical practice and b) participants benefitting from an excellent cosmetic result in the cream group are unlikely to want a biopsy scar. If, however, the clinician is concerned there is a recurrence, then he/she will biopsy that lesion exactly as would happen in routine follow-up care from other treatment modalities.

**Adverse events **will be collected from all participants up to the 1 year visit, thereafter only events considered serious or related to the trial comparators (imiquimod or surgery) will be collected.

### Health service research issues

An economic evaluation will include:

1. A cost-comparison analysis (net impact of all resource changes) of the two treatment strategies.

2. A cost-effectiveness analysis presented in the form cost per 'cleared participant'.

3. A valuation (in monetary terms using willingness to pay) of the perceived benefits of both treatment strategies, which (depending on results) could be incorporated into a cost-benefit analysis.

### Follow-up of outcome measures

**Figure 1:**
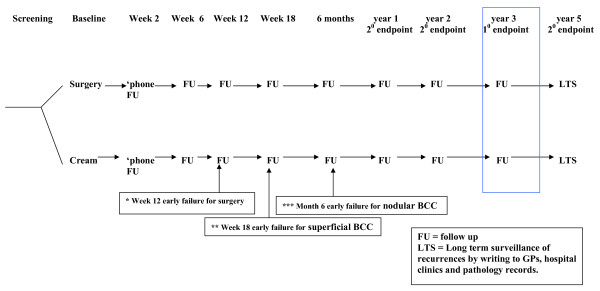
**SINS study design overview**.

The nurse will make a telephone call to the participant at two weeks to discuss any early problems, followed by clinic follow-up at 6, 12, and 18 weeks, 6 months, 1, 2 and 3 years. The dermatologist will also see the participant briefly, at 12 weeks (surgery only), 18 weeks (superficial, imiquimod only), 6 months (nodular, imiquimod only), 1, 2 and 3 years. A postal follow-up of documented recurrences at 5 years will be conducted by writing to GPs, hospital clinics and obtaining pathology records. See figure 1.

Extra clinic visits can be arranged if participants are concerned about adverse reactions or progress. Those participants developing a brisk inflammatory response to imiquimod to the extent that they find it difficult to continue can follow a pre-defined schedule of rest periods and reduced dosing frequency. These participants will be analysed with the main data with an appropriate sensitivity analysis.

### Randomisation

One lesion per patient will be chosen for the study, so that the unit of analysis will be patients rather than tumours. For those patients with multiple suitable BCCs, the research nurse will pick one *before *randomisation as below:

• The one that the patient is most bothered about, or first went to the doctor about

• If that does not apply, or the tumour in question does not meet the criteria, then the one that is easiest for the patient to reach.

• If this applies to more than one, the biggest will be chosen.

• If the patient wants both nodular and superficial BCCs treated, then a nodular BCC should be chosen to ensure there is enough cream for all (i.e. 12 weeks rather than six weeks of cream). If it happens that the histology shows the chosen nodular lesion not to be a BCC, but a superficial one is (assuming it had a biopsy), then the patient should be withdrawn, and re-randomised by the superficial BCC list.

Other current BCCs may be treated in the same way as the randomised one if the patient wishes, but this option should only be offered if needed, and BCCs are clinically suitable.

The identified BCC will be biopsied (three centres biopsy post randomisation, this being part of surgery for the surgery group). Patients eligible for inclusion and for whom consent has been obtained will be randomised to topical imiquimod or surgery, according to a pre-prepared randomisation schedule, generated by computer at the Trent Research and Development Support Unit (Trent RDSU). The allocation is obtained at the baseline visit, using a central telephone randomisation service run by independent staff at the Trent RDSU. This ensured concealment of allocation.

Randomisation will be stratified according to lesion type (nodular or superficial, defined clinically) and by centre, thus minimising the differences in the most important predictor baseline variables. There will be no attempt to equalise numbers of nodular and superficial BCCs randomised.

The participant's general practitioner and consultant will be informed about the participant's involvement in the study.

To minimise selection bias the allocation sequence will be concealed from participants, healthcare staff and investigators - the allocated treatment only being obtained after the participant had decided to take part in the trial (though the participant is always free to withdraw from the study at any time). Masking of the two very different interventions will not be possible for participants, and only partially possible for observers. We will determine whether there is evidence of dermatologists' observer bias at the 3 year visit by asking an independent panel of 3 dermatologists to judge "clinical success" from digital photographs.

The trial and full protocol are registered with the Cochrane Skin Group Ongoing Trials Register [http://www.nottingham.ac.uk/ongoingskintrials/ NB protocol not accessible from Cochrane], and with the UK meta-register of trials. Consumers with BCC have already been and will continue to be involved in commenting on the study design and conduct.

### Sample size

Based on initial Phase II data, it is highly unlikely that imiquimod cream will be superior to excisional surgery, so this is essentially a *non-inferiority study *i.e. the imiquimod success rate will be no worse than a pre-determined lower acceptable level.

The original total sample size estimate was for 740 participants, to be recruited over 18 months in three centres. This assumed a 1-tailed significance with (alpha) = 1%, power (1-β) = 80%, a 3 year success rate with surgery of 97% and for imiquimod of 90%, a lower 98% confidence limit of 84%, and allowed for a 10% drop-out rate. An imiquimod success rate of 90% was generally considered the lowest percentage to *change dermatologists' practice*, assuming success rates approaching 90% for other commonly used treatment modalities such as cryotherapy or curettage. The estimate is conservative, considering imiquimod as a direct competitor to surgery or other treatments, as opposed to using the cream first and then surgery if it fails i.e. sequential technology application.

Due to recruitment being slower than expected (many BCCs in high risk areas and the trial was too time consuming for potential participants), nine more centres were added and the recruitment period extended to 42 months; later (March 2006) the sample size was revisited. Various scenarios were considered. For a sample size of 500 the lower confidence interval would be within less than 10 percentage points of the actual imiquimod success rate assuming imiquimod success rate is at least 70% - probably acceptable precision for influencing practice. The revised overall sample size of 500 is considered to be both useful and achievable.

### Statistical analysis

The primary efficacy analysis will be performed using both a Full Analysis Set and the Per Protocol Set of participants. All other efficacy analyses will use the Full Analysis Set only. Data will be analysed using Stata version 10.1. No adjustments will be made for multiple endpoints. There is only one primary outcome variable. Secondary outcome variable findings and subgroup analysis findings will be interpreted with caution in view of the number of statistical tests undertaken.

Participants in the three centres performing the biopsy post-randomisation, and who were found not to have a BCC, did not continue in the study; they will be excluded from the analysis.

An independent Data and Safety Monitoring Committee reviewed data with the trial statistician relating to severe skin reactions.

**An early stopping rule** was proposed to safeguard participants with nodular BCC against unacceptably low early clearance rates from imiquimod however, due to the higher proportion of superficial BCCs than expected, all participants were recruited before the planned number of participants for the interim look were recruited, so the assessment was not made.

**Sub group analyses** are to be performed only for the primary outcome measure. These will assess whether the effectiveness of imiquimod cream in comparison to surgery is different in participants with:

• nodular lesions and those with superficial BCCs (confirmatory analysis)

• trunk lesions and those with head lesions (exploratory analysis)

• lesions ≤15 mm diameter and those with lesions greater than 15 mm diameter (exploratory analysis)

For each of the above analyses sensitivity analyses will be performed including and excluding participants with BCCs who are immunosuppressed at baseline.

**Missing data:** sensitivity analyses assuming both worst and best case scenarios will be performed for the primary outcome measure. **Potential outliers** will be identified by range checks and subjected to standard query generation and resolution. In order to **minimise loss of efficiency** in the analysis, the nine smallest centres will be pooled together to form a composite centre.

**Non inferiority margin:** This is based on a 97% success rate for surgery and a lower 98% confidence boundary of 84% for Imiquimod cream. This gives a non-inferiority margin (lower boundary of a 98% confidence interval for the relative difference expressed as a relative risk (imiquimod relative to surgery)) of 0.87. This margin was determined by the clinical judgement of the 4 clinicians who sit on the Trial Steering Committee. The non-inferiority margin will only apply to the analysis of the primary outcome for which non-inferiority is hypothesized.

Two-sided 98% confidence intervals for all outcomes will be presented. Results will be declared significantly "non-inferior" (primary outcome only) if the lower 98% confidence interval for the imiquimod effect relative to surgery is greater than the non-inferiority margin (0.87). Greater confidence will be placed on the results (primary outcome only) if the conclusions from the intention to treat (ITT) and per protocol (PP) analysis are consistent.

Data analysis will remain blinded until all data analysis has been performed and has been checked and approved by the Data Monitoring Committee.

#### Primary outcome measure

The number and percentage of participants successfully treated at 3 years in each treatment group, the absolute difference in percentages between groups and the corresponding 98% confidence interval for the absolute difference in percentages will be reported. The relative risk will be used to indicate the risk of being treated successfully in the imiquimod group relative to the surgery group. Poisson regression with a robust error variance will be used to estimate treatment effect, with centre and BCC type (fixed effects) included as covariates. This approach has been shown to be a reliable method to use for estimating adjusted relative risks for prospective studies with binary outcome variables [16].

Two separate analyses will be undertaken:

i) Adjusting for centre and tumour type

ii) Adjusting for centre, tumour type, tumour size, tumour site and immunosuppression.

#### Secondary outcome measures

**Clinical success **at 1, 2 and 5 years will be analysed and presented in the same way as for the primary outcome variable.

**Time to first recurrence (five categories):** Comparison of treatment groups will use ordinal regression analysis, adjusting for centre and BCC type (fixed effects). The number and percentage of participants in each of the 5 time of first recurrence categories will be reported by treatment group. The odds ratio arising from the continuation ratio model and the corresponding 98% confidence interval will be reported.

**Cosmetic appearance** of lesion site at 6 months and at 3 years as rated by the participant and by an independent dermatologist. The analysis of primary interest will be the participants rating at 6 months, focusing on head and neck, and then on all sites. The analysis of the dermatologist's ratings will be based on lesions at all sites. For each of the cosmetic appearance outcome measures, the number and percentage of participants with a lesion of excellent or good appearance in each treatment group, the absolute difference in percentages between groups and the corresponding 98% confidence interval for the absolute difference in percentages will be reported. The relative risk of having a lesion of excellent or good appearance in the imiquimod group relative to the surgery group and the corresponding two-sided 98% confidence interval will be presented. The inclusion of baseline variables in the model will be explored.

**Level of pain:** for each of the treatment period and the 16 week follow-up period, the number and percentage of participants who experience the following levels of pain (moderate or more severe, mild-moderate or less severe pain, no pain) will be reported by treatment group. The median number of days of moderate or more severe pain will also be reported.

**Cost effectiveness** for the different treatment modalities will be explored.

**Treatment compliance** in the imiquimod group will be reported. The amount of imiquimod cream used will be estimated from the daily diary (number of days of exposure). Insufficient exposure to imiquimod cream will also be reported (<4 weeks treatment for superficial BCCs; <8 weeks treatment for nodular BCCs), also the number unable to complete the full course as a result of an adverse event. In the surgery group treatment compliance will be the receipt of excisional surgery.

#### Analysis of safety and tolerability

Participants will be analysed according to the treatment they received, using descriptive statistics.

Full details of the analysis are given in a separate statistical analysis plan.

## Discussion

i) For *large *(greater than 4 cm diameter) superficial BCCs, an efficacy of around 90% might make imiquimod the treatment of choice because of better cosmetic results despite being slightly inferior to excisional surgery

ii) For smaller superficial BCCs, an efficacy of around 90% could still make it a more attractive option to non-surgical treatments with similar efficacy such as cryotherapy or curettage because it can be used at home

iii) Efficacy rates of as low as 70% for nodular BCC at low-risk sites could still be useful and cost-effective for dealing with the bulk of BCCs. These are non life-threatening lesions which can be dealt with surgically if recurrences occur. In other words, a "treat with the cream first and see what's left policy" might become a viable and more cost effective future treatment option.

This summary paper is based on version 9 of the study protocol, dated 27/07/06, and the analysis plan as at March 2010. A copy of the full protocol and analysis plan are available on request.

## Abbreviations

BCC: basal cell carcinoma; RCT: randomised controlled clinical trial.

## Competing interests

The authors declare that they have no competing interests.

## Authors' contributions

MO is responsible for day-to-day running of the trial and data collection/management, has been responsible for most revisions of the full protocol and wrote this summary (based on the full protocol), incorporating comments and suggestions from FB-H, HCW and SA. HCW is chief investigator for the study, and together with FB-H and others helped to design the study and write the original draft of the protocol, has commented on subsequent revisions of the full protocol and this summary. SA is the study statistician, has commented on revisions of the protocol, and has written the study analysis plan having particular responsibility for the analysis. FB-H conceived of the study, is the grant holder, helped to design the study and write the first draft of the full protocol, has commented on subsequent revisions of the full protocol and this summary. All authors have read and approved the final manuscript.

## References

[B1] Preston DS Stern RS Nonmelanoma cancers of the skin N Engl J Med 1992 327 1649 1662 1435901 10.1056/NEJM199212033272307

[B2] Miller SJ Biology of basal cell carcinoma (part1) J Am Acad Dermatol 1991 24 1 13 10.1016/0190-9622(91)70001-I 1999506

[B3] Telfer NR Colver GB Bowers PW Guidelines for the management of basal cell carcinoma Br J Dermatol 1999 141 415 423 10.1046/j.1365-2133.1999.03033.x 10583044

[B4] Wallberg P Skog E The increasing incidence of basal cell carcinoma Br J Dermatol 1994 131 914 915 10.1111/j.1365-2133.1994.tb08607.x 7857854

[B5] Bath-Hextall F Leonardi-Bee J Smith C Meal A Hubbard R Trends in incidence of basal cell carcinoma; Additional evidence from a UK-primary care database study International Journal of Cancer 2007 121 9 2105 2108 10.1002/ijc.22952 17640064

[B6] Van Hattem S Aarts MJ Louwman WJ Neumann HA Coebergh JW Looman CW Nijsten T de Vries E Increase in basal cell carcinoma incidence steepest in individuals with high socioeconomic status: results of a cancer registry study in The Netherlands Br J Dermatol 2009 161 4 840 845 10.1111/j.1365-2133.2009.09222.x 19438849

[B7] Bath-Hextall FJ Perkins W Bong J Williams HC Interventions for basal cell carcinoma of the skin Cochrane Database of Systematic Reviews 2007 1 CD003412 DOI: 10.1002/14651858.CD003412.pub2 10.1002/14651858.CD003412.pub2 17253489

[B8] Roenigk RK Ratz JL Bailin PL Wheeland RG Trends in the presentation and treatment of basal cell carcinomas J Dermatol Surg Oncol 1986 12 860 865 3734238 10.1111/j.1524-4725.1986.tb01993.x

[B9] McCormack CJ Kelly JW Dorevitch AP Differences in age and body site distribution of the histological subtypes of basal cell carcinoma: a possible indicator of differing causes Arch Dermatol 1997 133 593 596 10.1001/archderm.133.5.593 9158412

[B10] Thissen MR Neumann MH Schouten LJ A systematic review of treatment modalities for primary basal cell carcinoma Arch Dermatol 1999 135 1177 1183 10522664 10.1001/archderm.135.10.1177

[B11] McGovern TW Leffell DJ Mohs surgery Arch Dermatol 1999 135 1255 1259 10.1001/archderm.135.10.1255 10522675

[B12] Wang I Bendsoe N Klinteberg CAF Enejder AM Andersson-Engels S Svanberg S Svanberg K Photodynamic therapy vs. cryosurgery of basal cell carcinomas: results of a phase II clinical trial Br J Dermatol 2001 144 832 840 10.1046/j.1365-2133.2001.04141.x 11298545

[B13] Rowe DE Carroll RJ Day CL Jr Long-term recurrence rates in previously untreated (primary) basal cell carcinoma: implications for patient follow-up J Dermatol Surg Oncol 1989 15 315 328 2646336 10.1111/j.1524-4725.1989.tb03166.x

[B14] Thorpe K Zwarenstein M Oxman A Treweek S Furberg C Altman D Tunis S Bergel E Harvey I Magid D A pragmatic explanatory continuum indicator summary (PRECIS): a tool to help trial designers J clin Epidemiol 2009 62 464 475 10.1016/j.jclinepi.2008.12.011 19348971

[B15] Marks R Gebauer K Shumak S Amies M Bryden J Fox TL Owens ML Imiquimod 5% cream in the treatment of superficial basal cell carcinoma: results of a multicenter 6-week dose-response trial J Am Acad Derm 2001 44 807 813 10.1067/mjd.2001.113689 11312429

[B16] Zou GA Modified Poisson Regression Approach to Prospective Studies with Binary Data Am J Epidemiol 2004 159 702 706 10.1093/aje/kwh090 15033648

